# Maternal obesity alters C19MC microRNAs expression profile in fetal umbilical cord blood

**DOI:** 10.1186/s12986-020-00475-7

**Published:** 2020-07-06

**Authors:** Jia Jing, Yingjin Wang, Yanmei Quan, Zhijie Wang, Yue Liu, Zhide Ding

**Affiliations:** 1 Department of Histology, Embryology, Genetics and Developmental Biology, Shanghai Key Laboratory for Reproductive Medicine, Shanghai Jiao Tong University School of Medicine, Shanghai, 200025 China; 2 Department of Obstetrics and Gynecology, Shanghai Eighth People’s Hospital, Shanghai, 200235 China

**Keywords:** microRNAs, Obesity, Epigenetics, Umbilical cord blood, Fetus

## Abstract

**Background:**

To determine if overweight/obese pregnant women have altered microRNA expression patterns in fetal umbilical cord blood that may affect the development of offspring.

**Methods:**

Umbilical cord blood samples were obtained from the fetuses of 34 overweight/obese and 32 normal-weight women after delivery. Next generation sequencing (NGS) analyzed their miRNA expression patterns. miRanda and TargetScan databases were used to predict the miRNAs’ target genes followed by analyses of Gene ontology (GO) and Kyoto Encyclopedia of Genes and Genomes (KEGG) to perform function grouping and pathway analyses. qRT-PCR verified the identity of differentially expressed miRNAs that were revealed in the NGS results.

**Results:**

There was a positive correlation between newborn body weight and pregestational BMI of pregnant individuals (*r* = 0.48, *P* < 0.001). One hundred and eight miRNAs were differentially expressed between the normal and overweight/obese groups, which target genes were enriched in the metabolic pathway. Five C19MC miRNAs (miR-516a-5p, miR-516b-5p, miR-520a-3p, miR-1323, miR-523-5p) were upregulated in the overweight/obese group. Target enrichment analysis suggests their involvement in post-embryonic development, lipid and glucose homeostasis, T cell differentiation and nervous system development.

**Conclusions:**

C19MC miRNA expression upregulation in the fetal circulation during the gestation of overweight/obese pregnant women may contribute to altered multisystem metabolic pathway development in their offspring.

## Introduction

Over the past few decades, obesity is increasingly becoming a prevalent worldwide health problem in the human population. Currently, one-fifth of the women are obese at time of conception [1]. Maternal overweight and obesity not only can cause pregnancy related complications such as gestational diabetes, hypertension and preeclampsia, but also establish a detrimental genetic, hormonal and biochemical environment for the developing embryo/fetus [2, 3]. Maternal pregestational-overweight is an independent factor linked with infant and adolescence overweight and abdominal obesity [4, 5]. The “programming” hypothesis was advanced which suggests that exposure to suboptimal maternal conditions in the uterus, such as gestational diabetes mellitus (GDM), preeclampsia and obesity are risk factors promoting disease in the offspring because they disrupt homeostatic control of development [6, 7]. This concept is gaining acceptance based on epidemiological studies and results obtained in animal models of obesity [8–10].

It is now apparent that epigenetics is a contributing factor that affects how dietary and lifestyle behavior influence gene expression patterns controlling development during and following delivery of the fetus [11]. Such control is mediated through changes in DNA methylation status, histone modifications, chromatin remodeling and microRNA expression patterns [12]. The makeup of umbilical cord blood provides insight into the milieu that a fetus is exposed to during its development. miRNAs are epigenetic modulators of gene expression levels. These short 21–25 nucleotides are non-coding RNAs and expressed in numerous bodily tissues. They bind to complementary mRNA sequences and either block translation or degrade the mRNAs to which they are bound. Therefore, characterizing the miRNA expression profiles of umbilical cord fetal blood samples provides insight into the role that this epigenetic modulator has in controlling the gene expression patterns determining fetal development.

To delineate a role for miRNAs in controlling fetal development, their expression profiles were analyzed in overweight/obese and normal umbilical blood samples to determine if overweight/obese condition is associated with altered miRNA expression. We focused on a cluster of miRNAs expressed on chromosome 19 because of its higher expression in the overweight/obese group. C19MC, primate- and placenta- specific miRNAs, is one of the largest miRNA clusters expressed on chromosome 19 in humans [13]. This locus is relevant for gaining insight into the role of miRNAs in controlling fetal development since it was suggested that changes in C19MC microRNA expression patterns provide potential biomarkers for pregnancy related complications, such as preeclampsia, gestational hypertension and intrauterine growth restriction (IUGR) [14–16]. Even though the incidence of some of these complications can also be impacted by maternal obesity, it is unknown if pregestational weight in pregnant women is related to placental C19MC miRNAs change, which in turn contributes to altering gene expression levels in their offspring.

In this study, next generation sequencing (NGS) showed that during pregnancy miRNA expression patterns in fetal umbilical cord blood are different in overweight and obese individuals from those in the control group. This is the initial report showing that fetal cord blood of the offspring from overweight/obese group had several C19MC miRNAs whose expression levels were higher than in a normal-weight group. The predicted gene targets of these miRNAs may in turn contribute to fetal development.

## Materials & methods

### Recruitment of subjects

A total of 66 overweight/obese (*n* = 34) and normal-weight (*n* = 32) pregnant Chinese women were selected for this study. All participants were recruited at the Shanghai Eighth Peoples’ Hospital in China at the first prenatal check-up before the end of the first trimester (< 12 weeks). Exclusion criteria included smokers, drinkers, preeclampsia, drug use, chronic hypertension, diabetes, major fetal anomaly, active human immunodeficiency virus (HIV), hepatitis C, multiple pregnancies, prescription medications, or fertility treatment. Since only a small fraction of pregnant women are obese in China, all pregnant women enrolled in this study were classified into two groups: 1) control group (18.5 kg/m^2^ < BMI < 25 kg/m^2^), 2) overweight/obese group (BMI ≥ 25 kg/m^2^).

The study was approved by the institutional review boards of Shanghai Eighth Peoples’ Hospital and performed in accordance with the Helsinki Declaration. All of its participants provided informed written consent and received a close prenatal follow-up, including clinical examinations, ultrasonograms, urine and blood tests. Maternal pregnancy information was selected strictly from medical records. Body mass was expressed in kilograms and body mass index (BMI) was calculated as body weight divided by height squared (kg/m^2^) based on the World Health Organization (WHO) classifications. All offspring were born between the 37th to 42nd weeks and their sexes were recorded and a calibrated scale measured weights shortly after birth.

### RNA isolation

Following delivery, fetal umbilical cord blood samples of approximately 2.5 mL were collected and immediately placed in PAXgene Blood RNA tubes (PreAnalytiX, Qiagen, Germany) for storage at -80 °C. The Paxgene Blood miRNA kit (Qiagen, Germany) was used to isolate total RNA from the whole fetal cord blood. Briefly, PAXgene Blood RNA tubes were equilibrated to room temperature for at least 2 h before the next procedure in order to ensure that the blood cells were completely lysed. RNA Tubes were then centrifuged for 10 min at 3000 to 5000 xg (15-25 °C) and RNA was extracted followed by silica membrane-based purification according to the manufacturer’s protocol. A NanoDrop 2000 spectrophotometer (Fisher Scientific, USA) was used to measure both the RNA concentration and purity. RNA samples were stored at -80 °C immediately until further use.

### Small RNA library construction and sequencing

Umbilical cord blood samples (control group = 7, overweight/obese group = 9) were sent to The Beijing Genomic Institute (BGI, Beijing, China) for next generation sequencing (NGS) analysis. Small RNA library construction and sequencing was performed as follows. Agarose gel electrophoresis (1%) and spectrophotometry (260/280 nm) confirmed RNA quality. RNA integrity was assessed using an Agilent Technologies 2100 Bioanalyzer (Agilent Technologies, Inc., Santa Clara, CA, USA). Small RNAs ranging from 18 to 30 nucleotides were isolated and purified. Subsequently, the 5′ and 3′ adaptors for additional sequencing were ligated, and the small RNAs were reversely transcribed, and amplified. Finally, the purified cDNA library was sequenced using an Illumina Genome Analyzer (BGI Biotechnology, Cambridge, MA, USA) by following the manufacturer’s instructions.

### Bioinformatics analysis of differentially expressed miRNAs

Raw sequencing read were obtained by using Illumina analysis software and processed using the following steps: remove low quality tags, remove tags with 5 primer contaminants, remove tags without 3 primer, remove tags without insertion, remove tags with poly A and remove tags shorter than 18 nt. After filtering, the clean tags were mapped to the reference genome and other sRNA database. Particularly, cmsearch was performed for Rfam mapping [17]. Finally, the small RNAs were compared with the known miRNAs using miRBase 20.0 (http://www.mirbase.org/). The software miRDeep2 was used to predict novel miRNA by exploring the secondary structure. The small RNA expression level is calculated by counting absolute numbers of molecules using unique molecular identifiers [18]. Differential expression analysis was performed using the DEGseq [19], Q value ≤0.001 and the absolute value of Log2Ratio ≥ 1 was employed as the default threshold to judge the significance of expression difference.

The target genes of miRNAs were predicted by using miRanda and TargetScan databases. To annotate gene functions, all target genes were aligned against the Kyoto Encyclopedia of Genes (KEGG) and Gene Ontology (GO) database. GO enrichment analysis and KEGG enrichment analysis of target genes were performed using phyper, a function of R. The *P*-value was corrected using the Bonferroni method, and a corrected *P*-value < 0.05 was taken as a threshold.

### Quantitative real time-PCR (RT-qPCR) analysis

For validation of the differential gene expression results derived from NGS analysis, 500 ng RNA was converted into cDNA by using the miScript Reverse Transcription kit (Qiagen) according to the manufacturer’s protocol. The RT-qPCR was performed with the miScript SYBR Green PCR kit (Qiagen) whose total volume was 20 μL per PCR reaction containing 10 ng cDNA. The cycling conditions included 15 min incubation at 95 °C, followed by 40 cycles at 94 °C for 15 s, 55 °C for 30 s and 70 °C for 30 s (Applied Biosystems 7500, Fisher Scientific, USA). Each miScript Primer Assay was purchased from Qiagen Company, meanwhile human RNU6B was used as endogenous control. The relative expression of miRNAs was calculated with the threshold cycle (CT) method 2^−∆∆Ct^ [20].

### Statistical analysis

Statistical analyses of demographic data were performed with IBM SPSS Statistics 22. Data are expressed as mean ± standard error of mean (SEM). Levene’s statistic was used to test for homogeneity of variance. A normal probability plot was used to check for distribution of the data. Continuous variables between two groups were compared by Student’s *t*-test, and Mann–Whitney’s U-test was used to compare data without normal distribution. Categorical data were compared using chi-square test. The correlation between pregestational BMI and newborn weight was performed using Pearson correlation coefficient and simple linear regression. The correlation between miRNA expression and newborn weight was performed using Spearmen correlation test. Significance level was set at *P* ≤ 0.05.

## Results

### Clinical characteristics during pregnancy of control and overweight/obese groups and newborns

**Fig. 1:**
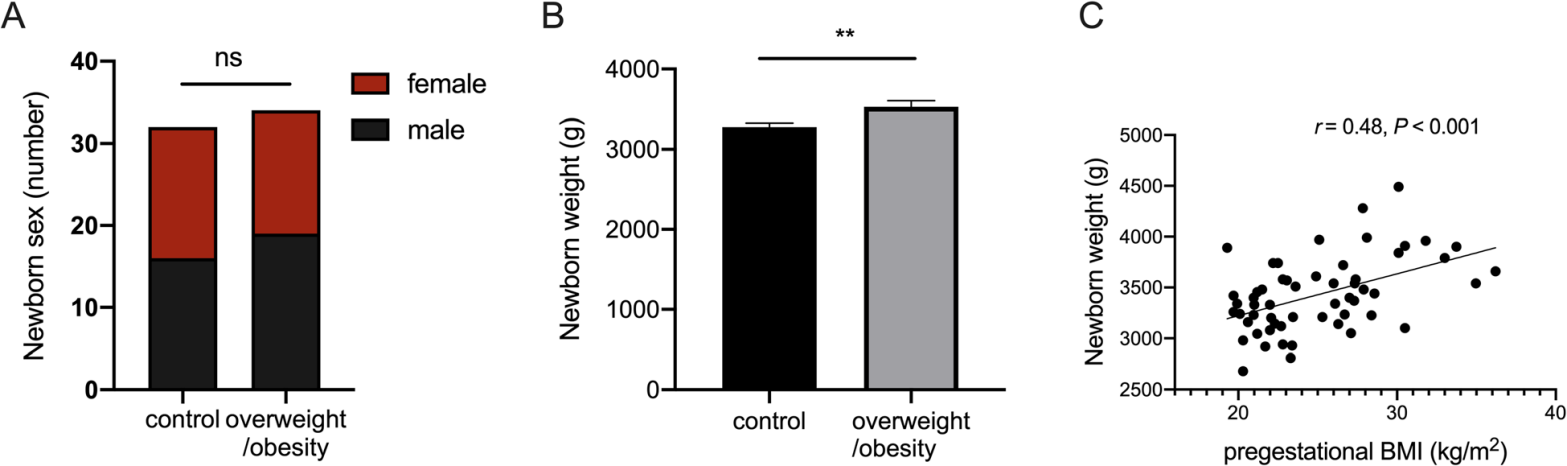
Comparison of newborn parameters between control and overweight/obese groups. **a** Numbers of male and female newborns in each group. NS: no significant difference. **b** Comparison of newborn weight between control and overweight/obese groups (*n* = 32, respectively). Data are expressed as mean ± SEM. **c** There is a positive correlation between newborn weight and the maternal pregestational BMI during pregnancy. (Pearson correlation coefficient, *r* = 0.48, *P* = 0.0002, *n* = 60). (**P < 0.01; ***P < 0.001)

A total of 66 umbilical cord blood samples were collected from fetuses in both normal-weight (*n* = 32, 18.5 kg/m^2^ < BMI < 25 kg/m^2^) and overweight/obese pregnant women (*n* = 34, BMI ≥ 25 kg/m^2^) soon after parturition without any complications. There were no significant differences in gestational weight gain between these two groups (12.66 ± 1.04 vs. 13.61 ± 0.95 kg, *P =* 0.510*)*, which excludes any influence of weight gain during pregnancy on miRNA expression profile changes. Meanwhile, neither maternal age nor frequency of cesarean delivery was different between two groups (Table 1). In newborns, ratio of sex in male or female was not different (Fig. 1a), whereas, newborn weight had increased more in the overweight/obese group than in the age-matched control group (3528 ± 76.29 vs. 3278 ± 52.00 g, *P* = 0.008, Fig. 1b). Moreover, the newborn weight had positive correlation with pregestational BMI of pregnant individuals (*r* = 0.48, *P* < 0.001, Fig. 1c), which indicates a potential metabolic change in fetuses exposed to maternal obesity in utero.

**Table 1:** Differences in clinical characteristics of pregnant women between control and overweight/obese groups

Subject characteristic	Control	Overweight/obesity	*P*-value
Pregestational BMI, kg/m2	21.28 ± 0.28	28.83 ± 0.55	< 0.001
Weight gain during pregnancy, kg	12.66 ± 1.04	13.61 ± 0.95	0.51
Maternal age, years	28.88 ± 0.65	29.47 ± 0.55	0.33
Cesarean delivery, n (%)	5 (16)	7 (20)	0.60
Cohort size NGS	7	9	
Cohort size RT-qPCR	25	25	

All parameters are expressed as n (%) or mean ± SEM. Student’s t-test (parametric continuous data) or Mann–Whitney’s U-test (nonparametric continuous data) determined significance of differences between the two groups. The chi-square test analyzed categorical data including cesarean delivery. *P* value of < 0.05 was considered significant. *BMI* body mass index

### Differential miRNA expression in fetal umbilical blood samples between overweight/obese and control groups

**Fig. 2:**
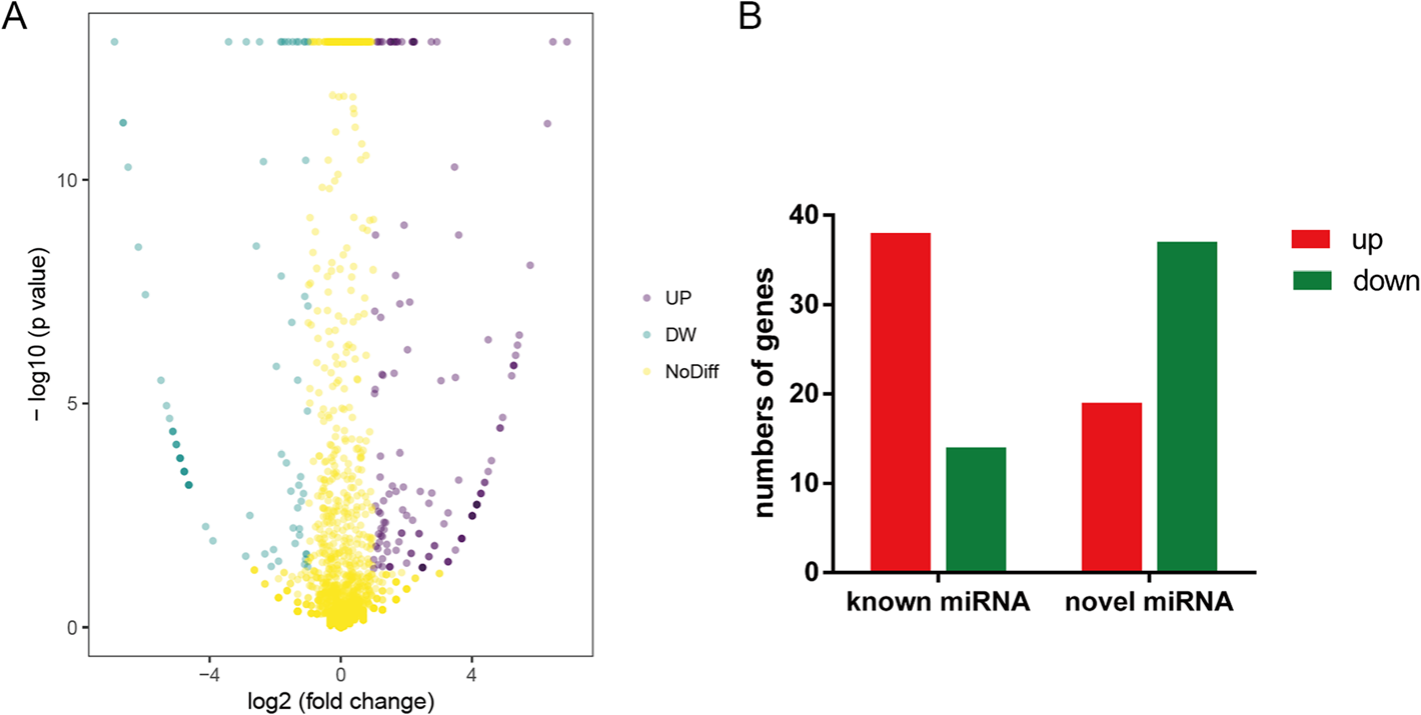
Analysis of differential miRNA expression in overweight/obesity and control groups. **a** Volcano plot illustrates the expression of different miRNAs detected by NGS. Purple dots represent up-regulated miRNAs and blue dots represent down-regulated miRNA with significant differential expression in overweight/obese pregnant group vs control group (|Log2| ≥ 1, Q value ≤0.001), and yellow dots represent genes with no significant difference. **b** The numbers of known as well as novel miRNAs that are either upregulated or downregulated and significantly different from one another

High-throughput NGS analysis was performed on fetal umbilical cord blood from 7 control and 9 overweight/obese pregnant women soon after giving birth. 1897 miRNAs were detected including novel and known human miRNAs. There were far fewer novel miRNAs (249/1897, 13%) than known human miRNAs, and most novel miRNAs had low expression levels. Among these miRNAs, there was a differential expression of 108 miRNAs between the control and overweight/obese groups (|Log2| ≥ 1, Q ≤ 0.001) (Fig. 2a). Moreover, among these differentially expressed miRNAs, 57 miRNAs were significantly up-regulated including 38 known miRNAs and 19 novel miRNAs, whereas 51 miRNAs were significantly down-regulated including 14 known miRNAs and 37 novel miRNAs (Fig. 2b) in the blood samples.

### Bioinformatic analysis of differentially expressed miRNAs

**Fig. 3:**
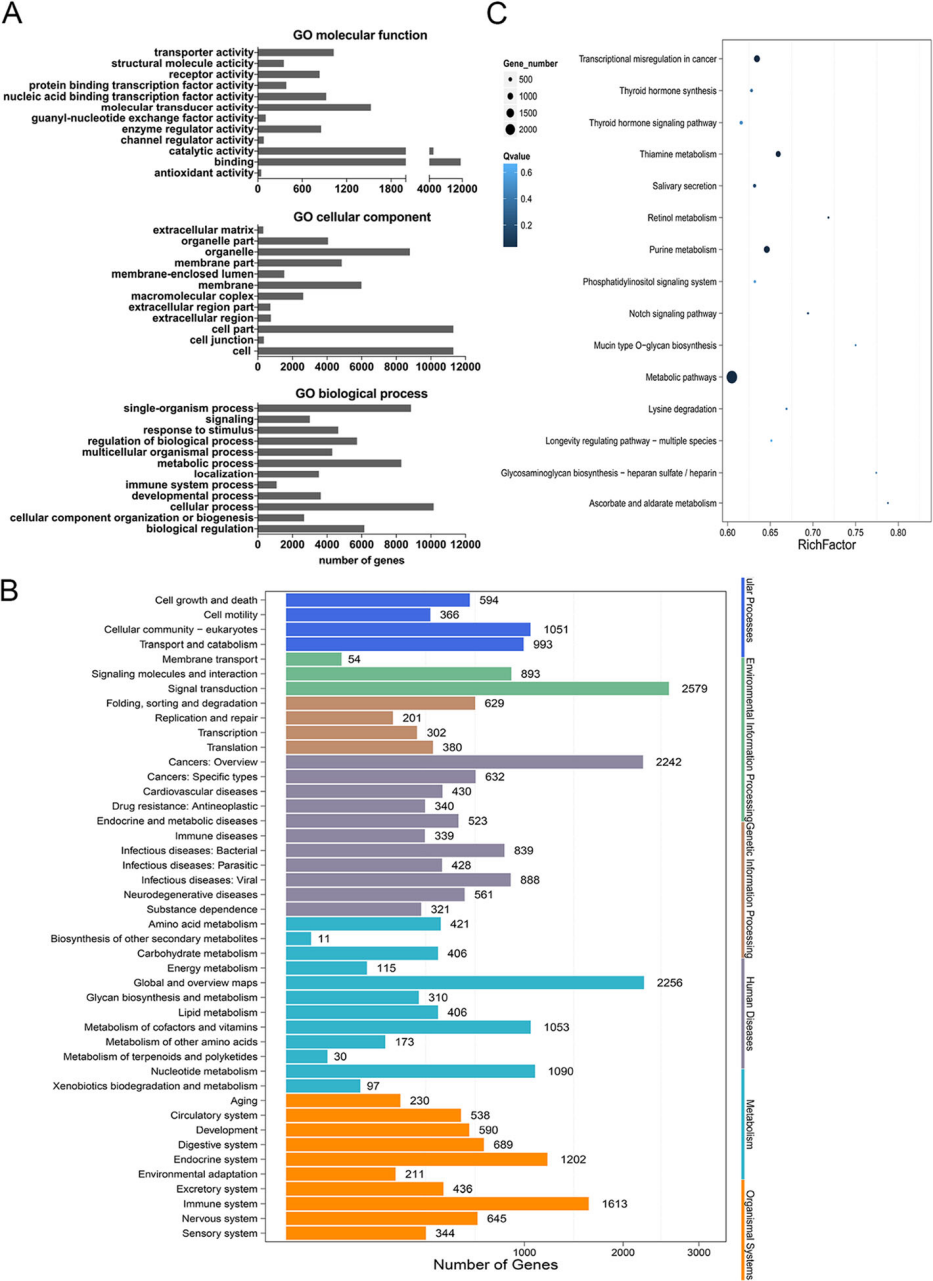
Bioinformatic analysis of differentially expressed miRNAs’ target genes. **a** GO analysis including molecular functions, cellular components and biological processes and **b** KEGG pathway analysis was performed on target genes of differentially expressed miRNAs between the control and overweight/obese groups. X axis represents numbers of target genes, and Y axis represents functional categories. **c** Scatter plot of KEGG pathway enrichment. X axis represents the enrichment factor, and Y axis represents the pathway items. The circle sizes are proportional with the number of genes, and their colors represent the significance (Q value: adjusted *P* value)

To explore the potential function of differential miRNAs, TargetScan and miRanda databases were employed to predict the target genes of these significantly different miRNAs and then GO and KEGG analysis of these target genes were performed. GO analysis includes characterizing their possible molecular function, cellular component and biological process involvement, which are shown in Fig. 3a. The analysis of molecular function of target genes revealed that they are mainly involved in binding, catalytic activity and molecular transducer activity. For the analysis of cellular component, the target genes are relevant to cell part, organelle and membrane***.*** The analysis of biological process shows that they participate in metabolic processes, responses to stimuli and developmental process (Fig. 3a). KEGG pathway analysis of target genes was classified into six terms, including cellular processes, environmental information procession, genetic information procession, human diseases, metabolism and organismal systems, and showed these genes mainly participated in endocrine, immune, digestive and nervous systems (Fig. 3b). Enrichment analysis showed target genes were enriched in metabolic pathway, which includes nucleotide, vitamin and cofactor, and lipid as well as glycan metabolism (Fig. 3c).

### C19MC miRNAs upregulation in fetal umbilical cord blood of overweight/obese group

**Fig. 4:**
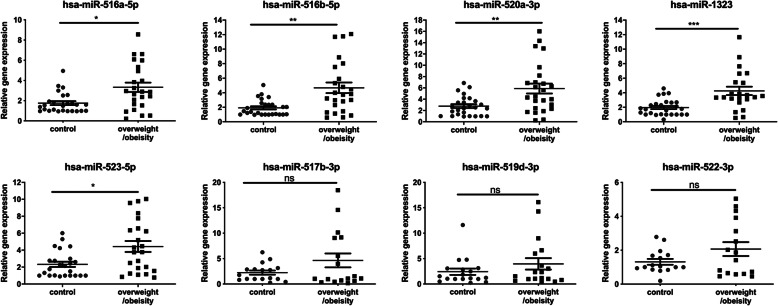
C19MC microRNAs were upregulated in the fetal umbilical cord blood of the overweight/obese groups. qRT-PCR validated the identity of eight differentially expressed C19MC miRNAs in NGS analysis. Values are reported as mean ± SEM. The results document that the relative gene expression levels of five C19MC microRNAs that are significantly up-regulated in the overweight/obese pregnant groups (*P < 0.05; **P < 0.01; ***P < 0.001; *n* = 25, respectively)

miRNAs with < 30 read counts were excluded in both groups because of low abundance. Among the 52 differentially expressed known miRNAs in fetal umbilical cord (Supplementary Table 1), eight of them originated from the C19MC miRNA cluster, which were also listed in the top 20-upregulated miRNAs in overweight/obesity group (Supplementary Table 2). qRT-PCR analysis was performed to validate the changes in expression levels of these identified C19MC miRNAs. Five miRNA expression levels were significantly different between the control and overweight/obese groups. Specifically, the hsa-miR-516a-5p expression level in the control and overweight/obese groups were 1.76 ± 0.19 and 3.32 ± 0.46 (*P* = 0.012), respectively. For the hsa-miR-516b-5p, its respective values were 1.92 ± 0.22 versus 4.68 ± 0.73 (*P* = 0.002). Hsa-miR-520a-3p had respective values that were 2.77 ± 0.34 versus 5.88 ± 0.88 (*P* = 0.004). The hsa-miR-1323 respective values were 1.96 ± 0.22 versus 4.26 ± 0.58 (*P* < 0.001). The hsa-miR-523-5p respective values were 2.33 ± 0.31 versus 4.43 ± 0.65 (*P* = 0.019). However, there were no differences in expression levels of either hsa-miR-519d-3p (2.42 ± 0.63 vs. 3.97 ± 1.12, *P* = 0.579), hsa-miR-517b-3p (2.25 ± 0.41 vs. 4.64 ± 1.37, *P* = 0.760) or hsa-miR-522-3p (1.32 ± 0.17 vs. 2.07 ± 0.41, *P* = 0.696) (Fig. 4). Moreover, newborn weight in pregnant individuals had positive correlations with miR-520a-3p and miR-523-5p expression (*r* = 0.33, *P* = 0.027; *r* = 0.33, *P* = 0.035, respectively) (Supplementary Fig. 1).

### GO analysis of target genes of five up-regulated C19MC miRNAs

Target gene prediction of the five up-regulated C19MC miRNAs revealed that 3549 target genes may be regulated by these miRNAs. Accordingly, these target genes were employed to perform GO analysis. The genes were grouped into 60 different subcategories with *P* values < 0.05. Among these subcategories, there was a significant enrichment in the following subcategories: positive regulation in phosphatidylinositol 3-kinase activity, post-embryonic development, memory, response to hydrogen peroxide, T cell differentiation, glucose homeostasis, MAPK cascade and nervous system development. These functional categories were placed within the immune, nervous systems, and lipid as well as glucose metabolic processes. GO name assignments of the predicted targeted genes of miRNAs involved in the relevant subcategories are listed in Table 2.

**Table 2:** GO analysis of predicted target genes of five validated C19MC miRNAs

Subcategory	GO name	numbers	*P*-value	Targeted mRNA
positive regulation of phosphatidylinositol 3-kinase activity	GO:0043552	15	0.0005	CCR7, FGR, SRC, TNFAIP8L3, ATG14, ERBB4, FGFR3, FLT1, IRS1, PDGFB, PTK2B, PTK2, TGFB1, VAV2, VAV3
post-embryonic development	GO:0009791	22	0.017	ALX4, MORC3, PLAGL2, PRDM1, SMAD2, SOX6, TAL2, TIPARP, ACVR2B, ALDH5A1, AGO2, CHST11, EMX1, GABRG2, HEG1, ITPR1, IMPAD1, KMT2A, PYGO1, SZT2, SELENOP, SOD2
memory	GO:0007613	19	0.023	HTR2A, CHRFAM7A, NTAN1, SHANK2, CNR1, DRD1, EIF4EBP2, FEN1, HRH1, HRH2, ITGA3, KALRN, PAK5, PLA2G6, PLCB1, KCNK10, KCNK4, PPP3CB, SLC6A4,
response to hydrogen peroxide	GO:0042542	16	0.032	BAK1, SRC, CASP3, DUSP1, GLRX2, GPX1, HP, HMOX1, MB, NR4A3, PPP1R15B, PTK2B, STK25, STAT1, SOD2, SDC
T cell differentiation	GO:0030217	11	0.033	CD3D, SOX4, SP3, CHD7, IL7R, NHEJ1, PREX1, PIK3CD, PPP3CB, PTPN2, PTPN22
glucose homeostasis	GO:0042593	27	0.034	ALMS1, CSMD1, HECTD4, HNF1A, SOX4, VGF, ADIPOR2, CNR1, CAV3, CRY1, CRY2, DHPS, FBN1, HNF4A, HIF1A, INPP5K, IRS1, INSR, NEUROD1, PRCP, PRKAA1, PRKAA2, PTPN2, RBP4, SLC2A4, SLC8B1, TCF7L2
MAPK cascade	GO:0000165	60	0.038	BRAP, CCL5, FYN, JAK1, NDST1, RASGRP3, RASGRP4, RASA1, RASA2, RAF1, RASGEF1A, SHC3, ACTN2, CAMK2B, CAMK2D, CAMKK2, CALM3, CSF2RA, CSF2RB, DOK4, DOK5, DUSP5, DUSP7, EGFR, EREG, ERBB3, ERBB4, FGF19, FGF7, FGFR3, GRB2, ITPKB, IRS1, IL17RD, IL2RB, IL31RA, KLB, LRRK2, MAPK4, MAPK8IP2, MAPKAPK3, MEF2D, NRG1, NF1, PDGFB, PSMD1, PSMD5, PSME3, PSME4, PSMA2, PSMB4, PTK2B, PTK2, RET, SPTA1, SPTB, SPTBN1, SPRED1, TGFB1, MYC
nervous system development	GO:0007399	67	0.038	MTR, DGCR14, GPSM1, RFNG, RBM45, ARHGEF7, SMARCC1, WNT8B, ZIC5, ATXN10, ATXN3, CHRM2, CHRM3, CHRDL1, CPLX2, CRIM1, DPYSL5, DOK4, DOK5, DYRK1A, EDNRB, ERBB4, FEZ1, FEZ2, FGF19, FUT10, FKTN, GBX2, GMFB, GRIK1, GPM6B, GAS7, HDAC4, IGSF9B, INSC, KALRN, LSAMP, MPPED2, MARK4, MAP1B, MBNL1, MEF2D, NRG1, NLGN1, NAV2, NR2E1, PRPS1, PCSK2, PPP2R5D, PCDHA10, PCDHA11, PCDHA2, PCDHA4, PCDHA5, PCDHA8, SEMA6A, SRRM4, SHOX2, SIGMAR1, SCN8A, TSHR, TMEM41B, TPP1, TMOD2, ZEB2,

Gene names and their numbers of predicted target mRNAs are listed in each GO subcategory. *P* value of < 0.05 was considered significant

## Discussion

In the current study, RNA deep sequencing was performed on fetal umbilical cord blood samples taken after parturition to determine if there is an association between maternal BMIs and fetal miRNA expression patterns. This assessment was undertaken since there are indications that increases in maternal BMIs are associated with altered fetal development and losses in offspring well-being that could be possibly attributable to changes in miRNA expression patterns. Such a possibility is tenable because miRNAs are epigenetic modulators that have an important role in the regulation of normal function and alterations in their expression patterns can underlie development of pathophysiological conditions in different disease states [21]. The rationale for this study is in line with studies showing that maternal miRNAs changes have been revealed to be related to maternal metabolism and fetal disease [22]. Altered expression of placental miRNAs in maternal serum has been implicated as potential biomarkers for fetal congenital heart defects [23]. Furthermore, some differences were identified in miRNAs expression profile patterns in the amniotic tissue between the obese and healthy pregnant women. These differences were associated with downregulation of neurotrophin, cancer/ErbB, mammalian target of rapamycin, insulin, adipocytokine, actin cytoskeleton expressions and mitogen-activated protein kinase signaling pathways [24]. Altered expression patterns of miRNAs in maternal blood and placental miRNAs were also identified in pregestational obesity and gestational obesity, which were associated with more adverse maternal metabolic status and precocious postnatal growth [25, 26]. Therefore, we undertook determining if there is an association between fetal miRNA expression patterns and maternal BMI since such an assessment has the potential to identify novel targets for treating abnormalities in utero that could later compromise offspring health and normal growth.

Umbilical cord blood samples were obtained noninvasively to evaluate if increases in maternal BMI impact on infant development through epigenetic changes such as miRNA expression. This is a credible question since a DNA methylation status change in fetal cord blood from obese pregnant women is correlated with maternal BMI, which alters fetal development through activating an involved inflammatory signaling pathway [27, 28]. In this study, NGS detected about 1897 miRNAs in control and overweight/obese groups. Out of all of these miRNAs, 94% of them (i.e.1789/1897) were not significantly different from one another in the overweight/obese and normal control groups. Conversely, 38 known and 19 novel miRNAs were significantly up-regulated, while 14 known miRNAs and 37 novel miRNAs were significantly down-regulated in the overweight/obese group. GO enrichments to clarify the identity of target mRNAs of these differently expressed miRNAs are mainly involved in the metabolic process (*n* = 8296), immune system process (*n* = 1085), regulation of biological process (*n* = 5734) and response to stimulus (*n* = 4639). KEGG pathway analysis showed that target genes were enriched mainly in the endocrine system (*n* = 1202), immune system (*n* = 1613), nervous system (*n* = 645), development (*n* = 590) and other systems. Moreover, target genes were mostly enriched in a metabolic pathway. Thus, we predict that these altered miRNAs may be relevant to epigenetic modification in metabolism, immunity, and responses to stimuli and other signaling pathways in fetus under exposure to maternal overweight and obesity.

C19MC is one of the largest miRNA gene clusters in humans. It maps to chromosome 19q13.41, and spans a ~ 100 kb long region [13]. They are primate-specific and comprise 46 miRNA genes [29]. This cluster is located within imprinted genes and it is only expressed by the paternally inherited chromosome [13]. Its expression is controlled by the methylation status in the upstream CpG rich promoter region at 17.6 kb in the C19MC cluster [30]. Tsai reported that the C19MC expression pattern was activated in human cancer cells through demethylation in a CpG-rich region, and this CpG-rich region is hypomethylated in the placenta tissue [31]. Notably, the C19MC members share common seed sequences and likely originate from a common ancestor [32]. Their expression are mainly restricted to the reproductive system and placenta [13], and some C19MC members are highly expressed in human embryonic stem cells (ESCs) [33]. Changes in this cluster have been hypothesized to affect human evolution and contribute to embryonic development [31]. Additionally, a fraction of these placenta-enriched miRNAs are released into the extracellular environment through exosomes, which were recently found to induce an antiviral immunity [34].

Interestingly, NGS results showed that eight C19MC miRNAs are among the 20 top up-regulated miRNAs in overweight/obese group, whereas none of the C19MC miRNAs were down-regulated in the current study. Some reports revealed that C19MC microRNAs play a role in cell proliferation, self-renewal, angiogenesis and pro−/anti-cancer activity [35, 36], but their biological action remains to be established. In this study, qRT-PCR validated that five C19MC miRNAs (hsa-miR-516a-5p, hsa-miR-516b-5p, hsa-miR-520a-3p, hsa-miR-1323 and hsa-miR-523-5p) were significantly upregulated in overweight/obese group. Predicted target genes of these five C19MC miRNAs showed their involvement in signaling pathways which contribute to the control of newborn development, such as the nervous system, post-embryonic development and lipid and glucose homeostasis signaling pathways. Therefore, these C19MC miRNAs may contribute to increased risks of asthma, autism and obesity in offspring induced by maternal obesity [37]. Moreover, several target genes listed in Table 2 (GRB2, HNF4A, IL2RB, INSR, KMT2A, NR2E1 and STAT1) were also identified in transcriptomic analysis of cord blood of maternal obesity [38], which agrees with the current study.

It is noteworthy that miRNAs analysis in this study was based on whole blood, which is a heterogeneous mixture of different blood cell types. However, the blood cell type is unknown from which C19MC miRNAs originate. Several C19MC miRNAs detected in the current study were previously described as being related to some pregnancy-related complications. For instance, circulating C19MC microRNAs (miR-516-5p, miR-517*, miR-518b, miR-520a*, miR-520 h, miR-525, and miR-526a) were found to be up-regulated in patients with established preeclampsia [16]. Furthermore, upregulation of some C19MC miRNAs (miR-516-5p, miR-517*, miR-520 h and miR-518b) is associated with a risk of later development of gestational hypertension [39], while seven placenta-specific microRNAs levels (hsa-miR-518b, hsa-miR-1323, hsa-miR-516b, hsa-miR-515-5p, hsa-miR-520 h, hsa-miR-519d, and hsa-miR-526b) from C19MC were significantly reduced in fetal growth restriction (FGR) placenta [15]. This commonality suggests that these obesity-induced rises in miRNA expression levels during pregnancy may serve as diagnostic markers of a higher risk of preeclampsia and gestational hypertension. In contrast to down regulation of some placenta-specific miRNAs in FGR, upregulation of these miRNAs in the overweight/obese group may explain why fetal exposure to maternal obesity results in a heavier body weight at birth than that in age-matched control groups.

Maternal obesity can induce macrosomia in the newborns [40], which is in accordance with our study that newborns from overweight/obese pregnant women were heavier than those in the control group. In the current study, the expression level of miR520a-3p and miR-520a-3p in fetal cord blood had positive correlations with newborn body weight. These results are also supported by a very recent study that lower levels of the placenta C19MC methylation status was associated with increased maternal pre-pregnancy weight, and with higher weight and fat mass of their offspring [41]. Therefore, it is plausible that declines in C19MC methylation status in the placenta of overweight/obese pregnant women upregulates the expression of C19MC miRNA, which adversely affects the gene expression pattern and phenotype of the fetus.

Ghaffari et al. reported that there is no difference in the fetal umbilical cord blood miRNA expression and placental miRNA expression in maternal obesity [42, 43]. Juracek performed TaqMan microarrays to identify five increased miRNAs in umbilical cord blood plasma from women with BMI > 25 (miR-1203, miR-143-3p, miR-582-5p, miR-510-5p, miR-450a-5p) [44]. Unlike the current study which employed NGS, these investigators instead isolated cell-free total RNA from umbilical cord blood, and determined miRNAs expression patterns with the Affymetrix miRNA GeneChip 3.0 Arrays or TaqMan microarray. This lack of agreement with our results is possibly attributable to the fact that circulating cell-free miRNAs in serum are different from those in whole blood. Furthermore, as a new generation sequencing technology, NGS provides a much more detailed and complete description of miRNA expression levels than gene microarray technology.

Besides, there are still some limitations in this research. The International Obesity Task Force recommended that the lower cutoffs are 18.5<BMI< 22.9 kg/m^2^ for normal range, BMI ≥ 23 kg/m^2^ for overweight, BMI ≥ 25 kg/m^2^ for obese I and BMI ≥ 30 kg/m^2^ for obese II in Asian people compared to that in Caucasian population, based on risk factors of type 2 diabetes and cardiovascular disease [45, 46]. Therefore, a lower cutoff for Asian population may be more relevant in the future clinical study to determine more accurately the role of maternal obesity on fetal development and offspring health during childhood and adulthood. Additionally, the results of the current study require validation by using a larger cohort in a future study.

## Conclusion

One hundred and eight differentially expressed miRNAs were detected and five C19MC miRNAs (hsa-miR-516a-5p, hsa-miR-516b-5p, hsa-miR-520a-3p, hsa-miR-1323 and hsa-miR-523-5p) had higher expression levels in fetal cord blood of overweight/obese pregnant women than in their normal counterparts. Bioinformatic analysis of these five C19MC miRNAs was consistent with suggested determinants underlying post-embryonic, fetal and newborns’ nervous system development and lipid and glucose homeostasis. Accordingly, these differentially expressed miRNAs may be potential diagnostic markers for detecting at an early stage abnormal fetal development in clinic.

## Supplementary information

**Additional file 1: Table S1.** Differentially expressed known miRNAs in overweight/obese and control groups.

**Additional file 2: Table S2.** Characteristics of eight differentially expressed C19MC microRNAs. List of microRNAs’ ID, NCBI location in chromosome and their nucleotide sequence.

**Additional file 3: Figure S1.** Correlation of C19MC expression and newborn weight. Correlation between the expression levels of hsa-miR-516a-5p, hsa-miR-516b-5p, hsa-miR-520a-3p, hsa-miR-1323 and hsa-miR-523-5p in fetal umbilical cord blood with newborn weight (*n* = 46). Spearmen correlation test was performed. The r value represents the degree of correlation, *P* value of < 0.05 was considered significant.

## Data Availability

The data generated or analyzed during this study are available from the corresponding authors on reasonable request.

## Abbreviations

BGI: Beijing genomic institute
BMI: Body mass index
ESCs: Embryonic stem cells
FGR: Fetal growth restriction
GDM: Gestational diabetes mellitus
GO: Gene ontology
HIV: Human immunodeficiency virus
IUGR: Intrauterine growth restriction
KEGG: Kyoto Encyclopedia of Genes and Genomes
miRNAs: MicroRNAs
NGS: Next generation sequencing
qRT-PCR: Quantitative real-time reverse-transcription polymerase chain reaction
SEM: Standard error of mean
WHO: World health organization
