# Supplementary figures and images for: Maternal obesity alters C19MC microRNAs expression profile in fetal umbilical cord blood
Source: Nutr Metab (Lond). 2020 Jul 6;17:52. doi: 10.1186/s12986-020-00475-7 (PMC7339545; doi:10.1186/s12986-020-00475-7)

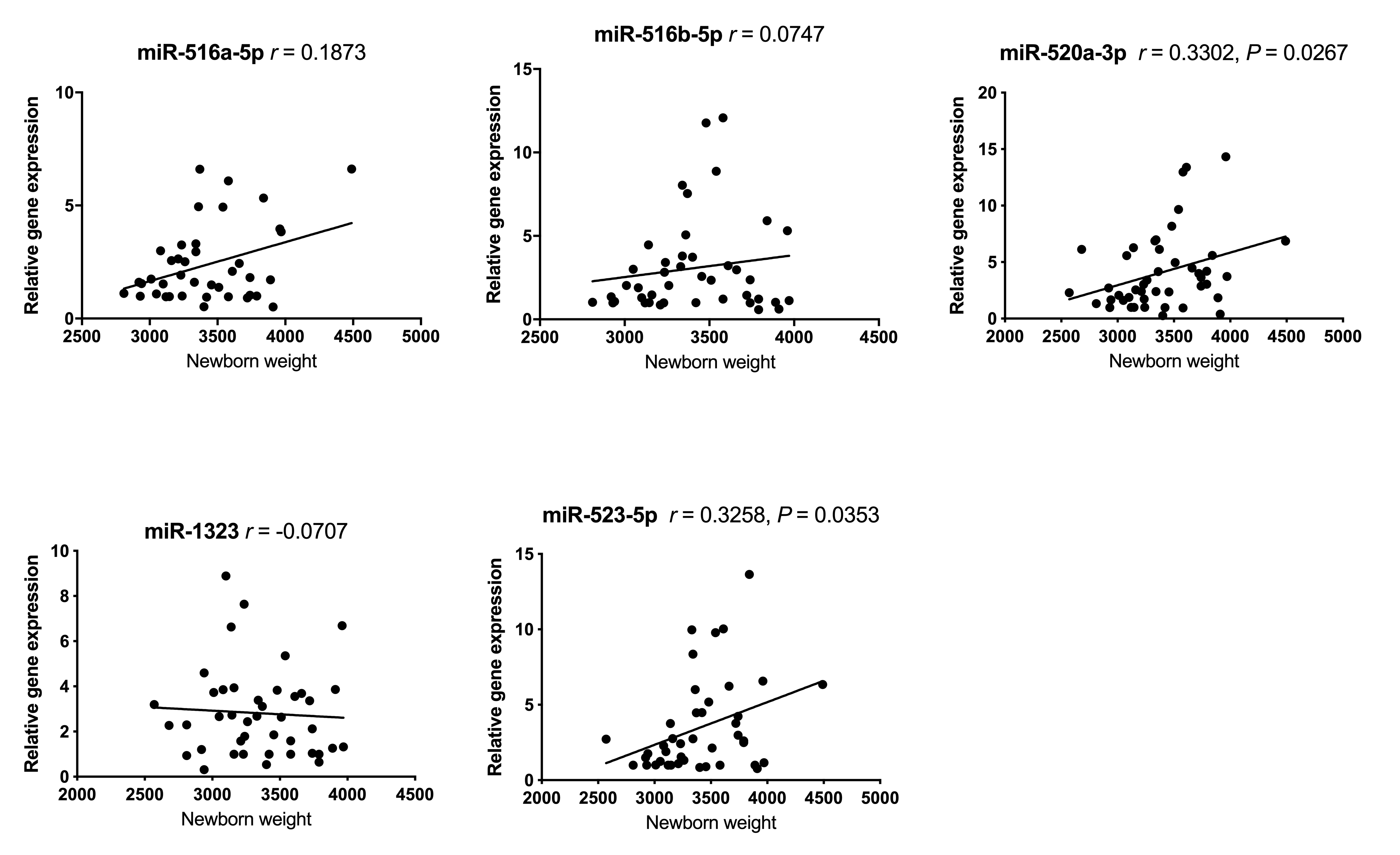

Supplement: Supplementary file 3 — Additional file 3: Figure S1. Correlation of C19MC expression and newborn weight. Correlation between the expression levels of hsa-miR-516a-5p, hsa-miR-516b-5p, hsa-miR-520a-3p, hsa-miR-1323 and hsa-miR-523-5p in fetal umbilical cord blood with newborn weight (n = 46). Spearmen correlation test was performed. The r value represents the degree of correlation, P value of < 0.05 was considered significant. [file 12986_2020_475_MOESM3_ESM.tiff]
